# Health-Related Quality of Life Scores and Values as Predictors of Mortality: A Scoping Review

**DOI:** 10.1007/s11606-023-08380-4

**Published:** 2023-08-31

**Authors:** Adriana G. Nevarez-Flores, Katherine J. Chappell, Vera A. Morgan, Amanda L. Neil

**Affiliations:** 1 Menzies Institute for Medical Research, University of Tasmania, Hobart, TAS Australia; 2 Neuropsychiatric Epidemiology Research Unit, School of Population and Global Health, The University of Western Australia, Crawley, WA Australia

**Keywords:** health-related quality of life, health states, utility, mortality, predictive model

## Abstract

Health-related quality of life (HRQoL) can be assessed through measures that can be generic or disease specific, encompass several independent scales, or employ holistic assessment (i.e., the derivation of composite scores). HRQoL measures may identify patients with differential risk profiles. However, the usefulness of generic and holistic HRQoL measures in identifying patients at higher risk of death is unclear. The aim of the present study was to undertake a scoping review of generic, holistic assessments of HRQoL as predictors of mortality in general non-patient populations and clinical sub-populations with specified conditions or risk factors in persons 18 years or older. Five databases were searched from 18 June to 29 June 2020 to identify peer-reviewed published articles. The searches were updated in August 2022. Reference lists of included and cited articles were also searched. Of 2552 articles screened, 110 met criteria for inclusion. Over one-third of studies were from North America. Most studies pertained to sub-populations with specified conditions and/or risk factors, almost a quarter for people with cardiovascular diseases. There were no studies pertaining to people with mental health conditions. Nearly three-quarters of the studies used a RAND Corporation QoL instrument, predominantly the SF-36, and nearly a quarter, a utility instrument, predominantly the EQ-5D. HRQoL was associated with mortality in 67 of 72 univariate analyses (92%) and 100 of 109 multivariate analyses (92%). HRQoL was found to be associated with mortality in the general population and clinical sub-populations with physical health conditions. Whether this relationship holds in people with mental health conditions is not known. HRQoL assessment may be useful for screening and/or monitoring purposes to understand how people perceive their health and well-being and as an indicator of mortality risk, encouraging better-quality and timely patient care to support and maximize what may be a patient’s only modifiable outcome.

**Supplementary Information:**

The online version contains supplementary material available at 10.1007/s11606-023-08380-4.

## INTRODUCTION

Quality of life (QoL) as a measure of a patient’s well-being or overall health began to be used for making medical decisions in the 1970s,^1^ particularly for survival decisions.^2^ In the 1990s, patients’ own evaluations of their health status came to be considered in relation to the QoL associated with health aspects of the disease and/or its treatment as perceived by the patient.^3^ The term health-related QoL (HRQoL) was then conceived.^3^ HRQoL is an important outcome with inherent value when treating patients or improving patient care. Assessing HRQoL can guide decision-making on treatments at population and patient levels,^4^ and predict the success of treatment.^1^

A variety of instruments have been developed for assessing HRQoL. Some instruments are generic (for use in populations or sub-populations, irrespective of illness or conditions), while others are disease specific (for use in populations with a specific disease).^1^ Further, some instruments allow for derivation of a score as a holistic or composite assessment of the individual’s health status, while other instruments employ multiple independent scales that are reported separately. For multi-attribute utility instruments, which provide generic and holistic assessments, a single score or “utility” is obtained through a preference-based assessment of health states determined from responses to multiple items and/or dimensions.

An association between HRQoL with mortality in the general non-patient population has been systematically reviewed.^5^ However, HRQoL was predominantly assessed as one of multiple possible attributes or dimensions of the individual’s life.^5^ An association between disease-specific assessments of HRQoL and mortality has been reviewed in patients with heart failure.^6,7^ The ability of utilities to predict future morbidity and mortality is also being explored. Clarke and colleagues^8^ reported that index scores derived from the EuroQol Five Dimensions questionnaire (EQ-5D) can be used to independently identify diabetic patients at higher risk of diabetic complications and death, while querying other instruments predictive abilities. Clarke et al. also found cumulative and/or extreme problems were important in identifying high-risk patients, a determination that could only be pursued through a holistic summary measure.

Despite the available evidence, information on the relationship between holistic assessments of health status assessed with generic instruments and mortality in both general non-patient populations and clinical sub-populations has not been reported. Therefore, based on these findings and queries, and previous work on HRQoL in people with psychotic disorders,^9,10^ a sub-population in which there is significant premature mortality,^11^ the aim of this scoping review is to map the evidence on generic, holistic assessments of HRQoL as predictors of mortality in general non-patient populations and clinical sub-populations. We also aim to ascertain conditions where this relationship has been examined, instruments and statistical methods employed, findings of those analyses, and in turn identify gaps.

## METHODS

The present scoping review follows the methodology of the Joanna Briggs Institute for scoping reviews^12,13^ and recommendations of the Preferred Reporting Items for Systematic Reviews and Meta-Analyses extension for scoping reviews (PRISMA-ScR).^14^ The protocol of the review was registered with the Open Science Framework (https://osf.io/vdqga) on 18 June 2020.

### Data Sources and Searches

Five electronic databases (CINAHL (via EBSCOhost), EMBASE (via Ovid), Science Direct, Web of Science, and PubMed) were searched from 18 to 29 June 2020 and updated in August 2022. Searches were limited to English language with no time restrictions; syntax, filters, and Boolean operators were employed. The search strategy for PubMed is presented in the Supplementary Table 1. Records obtained were exported to EndNote X8 and duplicates deleted. The reference lists of selected articles and literature cited in the “Introduction” section were searched to identify additional articles.

### Study Selection

Titles and abstracts of identified studies were screened by two independent reviewers (A.N.F. and A.N.) with full texts of selected articles and then assessed for eligibility. For inclusion, studies needed to be peer-reviewed published articles that considered generic HRQoL scores as predictors of mortality in the general population and/or sub-populations with specified conditions or risk factors, in persons aged 18 years or older. HRQoL scores needed to be assessed with a recognized and complete HRQoL generic instrument, and a holistic score provided. For instruments that assessed mental and physical components separately, both summary scores needed to be provided. Methods for the collection/reporting of mortality needed to be specified. Further, the methodology used for the assessment of predictors had to be detailed and the findings of whether HRQoL scores predicted mortality clearly stated. Studies could be conducted in any contextual setting except prison, and in any geographic location. Study designs included were as follows: case-control; cross-sectional with follow-up; retrospective and prospective cohort; randomized and non-randomized controlled trials; and quasi-experimental studies. Systematic reviews and meta-analysis were excluded.

### Data Extraction and Synthesis of Evidence

The data charting form was developed by A.N.F. and A.N.; then, A.N.F. charted the data under the guidance of A.N. During extraction, results were discussed by the two reviewers and the data charting form modified to include other items of relevance to this review. Inconsistencies and disagreements were resolved by consensus; when that was not possible, K.J.C. acted as a third reviewer. Variables identified for extraction a priori were as follows: author(s), year of publication, country of origin, study population, sample size, design, instruments used, statistical method, and key findings (by type of analysis: univariate, multivariate), and during extraction: age, sex, and timeframe (i.e., follow-up). Univariate analyses were included as “proof of concept” of the overall association between HRQoL and mortality. Multivariate analyses were included as evidence of an independent association after adjustment for a range of other factors. Data extracted from each article were grouped with reference to the study population on the basis that health conditions should be given primary consideration when assessing predictors of mortality. In justification, in 2019, nearly half of global deaths (44%) and four-fifths (80%) of the top ten causes of death were due to noncommunicable causes.^15^ Given the heterogeneity of the results and the objective of this scoping review, a narrative synthesis was then undertaken to synthesize the findings of included studies. Specifically, a descriptive summary of studies is provided to address the review’s aims.^12^

## RESULTS

**Fig. 1:**
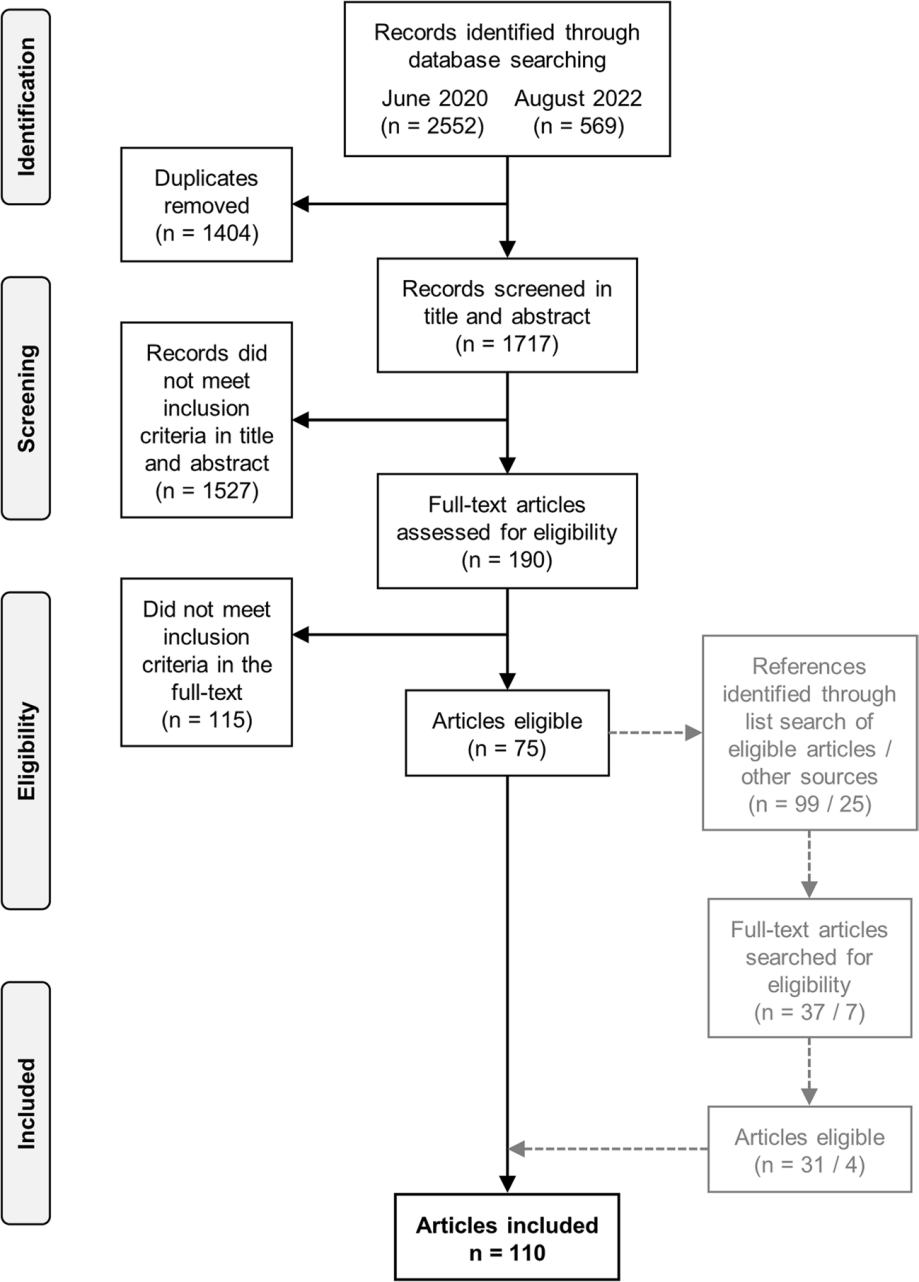
Prisma diagram of study selection.

In June 2020, 2552 records were identified through the database searches; an additional 569 records were identified in August 2022. Of a total of 3121 records, 1404 were duplicates and deleted. Title and abstract of 1717 records were then screened with 190 identified as requiring full-text review. Of these 190 articles, 115 did not meet inclusion criteria, including 54 that used a disease-specific instrument to measure HRQoL and 21 that did not employ holistic assessments; 76 articles were thus eligible for inclusion (Fig. 1). In a final step, 99 references from reference lists of eligible articles and 25 references identified within articles cited in the “Introduction” section of this study were examined, with the full-text obtained and reviewed for 37 and 7 respectively, with 31 and 4 identified as eligible. Thus, 110 articles were included in this review, for which the general characteristics and descriptive summary are presented in Tables 1 and 2, respectively.

**Table 1:** General Characteristics of Included Articles (*N*=110)

Characteristic	Number	(%)
Publication year		
≤2000	2	(1.8%)
2001–2005	14	(12.7%)
2006–2010	37	(33.6%)
2011–2015	26	(23.6%)
2016–2020	25	(22.7%)
2021–2022	6	(5.5%)
World region of publication*		
Europe	42	(38.2%)
North America	47	(42.7%)
South America	1	(0.9%)
Western Pacific	9	(8.2%)
Various countries	9	(8.2%)
Not available	2	(1.8%)
Study population		
General population	7	(6.4%)
Older persons (including veterans)	16	(14.5%)
Postmenopausal women	1	(0.9%)
*Clinical sub-populations*		
Admitted to intensive care/emergency departments	9	(8.2%)
Cancer-related	14	(12.7%)
Cardiovascular diseases	24	(21.8%)
Dementia	1	(0.9%)
Diabetes	4	(3.6%)
Human immunodeficiency virus	1	(0.9%)
Kidney diseases, dialysis, and haemodialysis	17	(15.5%)
Liver diseases	2	(1.8%)
Musculoskeletal	5	(4.5%)
Neurological disorders	2	(1.8%)
Respiratory diseases	6	(5.5%)
Systemic lupus erythematosus	1	(0.9%)
Instrument employed^†^		
RAND Corporation QoL surveys	86	(78.2%)
EuroQol five dimensions questionnaire (EQ-5D)^‡^	20	(18.8%)
EQ-5D Visual Analogue Scale	18	(16.7%)
Health Utilities Index Mark 3	2	(1.8%)
Minimum Data Set Health Status Index	1	(0.9%)
Nottingham Health Profile	1	(0.9%)
The 15-Dimensional instrument	1	(0.9%)
Statistical methods (univariate analyses)		
Cox proportional hazards regression	43	(39.1%)
Logistic regression	17	(15.5%)
Kaplan–Meier estimator	9	(8.2%)
Gehan generalized Wilcoxon test	1	(0.9%)
Linear regression	1	(0.9%)
Log rank test *P* value	1	(0.9%)
Non specified regression	1	(0.9%)
Not provided	37	(33.6%)
Statistical methods (multivariate analyses)		
Cox proportional hazards regression	79	(71.8%)
Logistic regression	25	(22.7%)
Fine and Gray competing risks regression	1	(0.9%)
Forecasting models§	1	(0.9%)
Logistic regression and Cox proportional hazards	1	(0.9%)
Non specified regression	1	(0.9%)
Proportional subdistribution hazards	1	(0.9%)
Not presented	1	(0.9%)
Univariate prediction of mortality		
No	5	(4.5%)
Yes (including those varying by model^‖^)	47	(42.7%)
SF physical component score	19	(17.3%)
SF mental component score	1	(0.9%)
Not presented	38	(34.5%)
Multivariate prediction of mortality		
No	9	(8.2%)
Yes (including those varying by model/instrument)	71	(64.5%)
SF physical component score	24	(21.8%)
SF mental component score	5	(4.5%)
Not presented	1	(0.9%)

*According to the World Health Organization regions

^†^Sixteen studies used two or more instruments

^‡^Included predicted EQ-5D

^§^Deep neural networks model, K nearest neighbor algorithm, support vector machine, naïve Bayes classifier, and Cox regression model

^‖^Some studies included different models in their analyses with variation in results

**Table 2:** Descriptive Summary of Studies Included in the Scoping Review Organized by Year of Publication

Study characteristics								Prediction of mortality by analysis			
Study^Reference^ year	Location	Sample size, *n*	Age*	Sex† (%)	Population	Instrument(s)	Timeframe‡	Univariate/comment		Multivariate/comment	
Deoreo^90^ 1997	USA	1000	58.2	50%	On dialysis	SF-36^§^	2.0	NA		Y	Only PCS
Rumsfeld et al.^79^ 1999	USA	2480	63	1%	After CABG	SF-36	0.6	Y	Only PCS	Y	Only PCS
Curtis et al.^65^ 2002	USA	1778	64.1	24.6%	After CABG	SF-36	2.0	Y	Only PCS	Y	
Fan et al.^38^ 2002	USA	10 947	67.8	3%	Veterans on primary care	SF-36	2.0	Y		Y	
Domingo-Salvany et al.^118^ 2002	Spain	312	65	100%	W/ COPD	SF-36	4.8	Y	Only PCS	Y	Only PCS
del Aguila et al.^114^ 2003	USA	180	61.3	46.1%	W/ ALS	SF-36	4.0	N		Y	Only PCS
Knight et al.^95^ 2003	USA	14 815	61	47.2%	On haemodial.	SF-36	1 & 2	NA		Y	
Lowrie et al.^99^ 2003	USA	13 952	59	48.6%	On dialysis	SF-36	0.6	NA		Y	
Fan et al.^28^ 2004	USA	7702	65.4	3.4%	Veterans on primary care	SF-36	1.0	Y		Y	
Lopez Revuelta et al.^98^ 2004	Spain	318	61.9/57.0^‖^	39%/40%^‖^	End-stage renal disease	SF-36	1 to 3	Y		Y	Varies by model
Sprenkle et al.^121^ 2004	USA	8354	65	4.4%	W/ asthma or COPD	SF-36	1.0	Y		Y	
DeSalvo et al.^26^ 2005	USA	21 762	64	3.6%	VA patient population	SF-36	1.0	NA		Y	Only PCS
Ho et al.^67^ 2005	USA	3160	64	≥65, 1.8% <65, 1.2%	After cardiac surgery	SF-36	0.5	NA		Y N	Older Young
Rodriguez-Artalejo et al.^45^ 2005	Spain	433	77.2	56%	Admitted to ED for HF	SF-36	0.22^¶^	Y		Y	Only PCS
Takaki et al.^104^ 2005	Japan	490	60.3	33.9%	On haemodial.	SF-36	3.0	Y		Y	Only PCS
Singh et al.^112^ 2005	USA	34 440	64.4	4%	W/ arthritis	SF-36	1.0	Y		Y	
Dorr et al.^27^ 2006	USA	3042	77.9	54.9%	Older persons	SF-12	2.3	Y		Y	
Park et al.^54^ 2006	South Korea	142	62.1	50%	W/ terminal Ca	EQ-5D	18.5 d^¶^	Y		Y	
Piotrowicz et al.^77^ 2006	USA	1058	NA	NA	W/ EF ≥30% post-infarction	SF-12	1.0	Y		Y	
Valdés et al.^106^ 2006	Spain	199	63.5	35.2%	On haemodial.	SF-36	1.0	NA		Y	Only MCS
Carusone et al.^116^ 2007	Canada	347	85.7/84.6^#^	69.2%	Respiratory infections	MDS-HSI	30 d	N		N	
Faller et al.^66^ 2007	Germany	231	64	29.40%	W/ CHF	SF-36	2.7	Y		Y	Varies by model
Fernandez et al.^124^ 2007	USA	552	36.8	89%	W/ SLE	SF-36, SF-6D	10.0	Y	SF-6D and SF-36 PCS	N	
Grignon et al.^52^ 2007	USA	571	59.7	32.9%	Head/neck Ca	SF-36	5.0	NA		Y	Only PCS
Hofhuis et al.^43^ 2007	Netherlands	451	71	38.8%	ICU admission	SF-36	0.5	NA		Y	
Kaplan et al.^22^ 2007	Canada	12 375	NA	52%	Canadian people	HUI3	9.0	NA		Y	
Lenzen et al.^73^ 2007	31 countries	3786	62.8/69**	24%/22%**	W/ CAD	EQ-5D, EQ-5D VAS	1.0	Y		Y	VAS, EQ-5D NA
Mathews and May^123^ 2007	USA	965	37	12%	HIV-infected adults	EQ-5D VAS	4.5	NA		Y	
Tsai et al.^33^ 2007	Taiwan	4424	NA	NA	Older persons	SF-36	3.0	NA		Y	
Esteban^119^ 2008	Spain	611	67.2	NA	W/ COPD	SF-36	5.0	NA		N	
Halpin et al.^120^ 2008	NA	1834	64	23%	W/ COPD	SF-36	1.0	Y		Y	
Karvonen-Gutierrez et al.^53^ 2008	USA	495	58	18.4%	Head/neck Ca	SF-36	5.1^¶^	Y	Only PCS	Y	Only PCS
Kroenke et al.^18^ 2008	USA	40 337	NA	100%	Healthy women	SF-36	4.0	NA		Y	
Steinberg et al.^81^ 2008	USA	1016	64	18%	W/ ventricular arrhythmias	SF-36	1.5 ± 1.0	Y	Only PCS	Y	Only PCS
Thombs et al.^84^ 2008	Canada	800	61.5	33.4%	W/ ACS	SF-12	1.0	Y	Only PCS	Y	Varies by model
Cella et al.^50^ 2009	USA	750	62/59^††^	28.5%	Renal cell Ca	EQ-5D VAS	28 d	NA		Y	
Clarke et al.^8^ 2009	Australia, Finland, New Zealand	7348	66.9	38%	W/ type 2 diabetes	EQ-5D	5.0	NA		Y	
Grande et al.^51^ 2009	UK	100	71.5/69.2^‡‡^	38.0%	Colorectal or lung Ca	SF-36	5.0	Y	Only MCS	Y	Only MCS
Hayashino et al.^93^ 2009	Japan	527	62.4	30.2%	On haemodial.	SF-36	1.9^¶^	NA		Y	Only PCS
McEwen et al.^87^ 2009	USA	7892	NA	54.1%	W/ diabetes	EQ-5D	3.7*	NA		Y	
Sacanella et al.^46^ 2009	Spain	230	74.5	39%	Non-elective ICU admission	EQ-5D VAS	3.2	Y		Y	
Zhang et al.^86^ 2009	USA	1785	53.4	41.20%	W/ CAD	SF-36	5.0	Y	Only PCS	Y	Only PCS
Ashing-Giwa et al.^49^ 2010	USA	353	51	100%	Cervical Ca survivors	SF-12	5.0	Y		Y	
Issa et al.^68^ 2010	Netherlands	503	67	27%	W/ PAD	EQ-5D	3.0	Y		Y	
Kao et al.^69^ 2010	Canada, USA	507	64.9	21.7%	ICD recipients	SF-36	1.0	NA		N	
Kusleikaite et al.^96^ 2010	Lithuania	183	56.7	43.7%	On haemodial.	SF-36	6.0	Y		Y	
Lacson et al.^97^ 2010	USA	44 395	61.2	46.0%	On dialysis	SF-36, SF-12	1.0	Y		Y	
Landman et al.^89^ 2010	Netherlands	1353	67.8	57.6%	W/ type 2 diabetes	SF-36	10.0	NA		Y	
Myint et al.^19^ 2010	UK	17 736	NA	56.2%	Norfolk residents	SF-6D	6.5^a^	NA		Y	
Otero-Rodriguez et al.^31^ 2010	Spain	2373	NA	57.6%	Older persons	SF-36	4 to 6	Y		Y	Varies by model
Peng et al.^101^ 2010	Taiwan	888	57.9	56.2%	On haemodial.	SF-36	7	Y	Only PCS	Y	Only PCS
Tanikella et al.^107^ 2010	USA	252	54	36%	W/ portal hypertension	SF-36	422^§§^	Y	Only PCS	Y	Only PCS
Zuluaga et al.^47^ 2010	Spain	416	75.3/78.4**	56%	ED admission	SF-36	7.0	Y		Y	Only MCS
Feroze et al.^91^ 2011	USA	705	53.5	47%	On haemodial.	SF-36	6.0	Y		Y	
Haring et al.^16^ 2011	Germany	4259	47.0/70.3**	51%	German citizens	SF-12	10.0	NA		Y	Only PCS
Jerant et al.^17^ 2011	USA	22 259	NA	53.1%	USA population	SF-6D, EQ-5D, pEQ-5D, EQ-5D VAS	1.0	Y		Y	
Muñoz et al.^20^ 2011	Spain	3724	54.1	51.9%	Spanish population	SF-12	6.3^¶^	Y	Only PCS	Y	Only PCS
Pedersen et al.^76^ 2011	Netherlands	870	62.6	27.8%	PCI patients	EQ-5D VAS	1.0	Y		Y	
Szekely et al.^82^ 2011	Austria, Canada, Colombia, France, Germany, Hungary, India, Israel, Italy, Mexico, Poland, Romania, Thailand, Netherlands, UK, USA	4811	NA	20.3%	After CABG	SF-12	4.0	NA		Y	Only MCS
Cavrini et al.^25^ 2012	Italy	5256	74.5	54.6%	Older persons	EQ-5D, EQ-5D VAS	2.0	Y	EQ-5D, VAS NA	Y	Both
Joyce et al.^94^ 2012	USA	439	NA	30%	W/ AKI	HUI3	1.0	Y		Y	
Osthus et al.^100^ 2012	Norway	252	60.2	34.1%	On dialysis	SF-36, SF-12	4.5	Y	Both PCS	Y	Both PCS
ter Horst et al.^83^ 2012	Netherlands	2501	65.3	21%	Underwent CABG	EQ-5D, EQ-5D VAS	30 d	N		NA	
Romanus et al.^59^ 2012	USA	267	NA	44%	Pancreatic Ca	EQ-5D, EQ-5D VAS	0.2	Y		Y	
Williams et al.^88^ 2012	Australia	9979	51	55%	W/ diabetes	SF-36	7.4	NA		Y	
Pompili et al.^57^ 2013	NA	131	68	21%	Lung Ca	SF-36	3.33^¶^	Y	Only PCS	Y	Only PCS
Saquib et al.^39^ 2013	USA	20 308	62.8	100%	Postmenopausal women	SF-36	3.0	Y		Y	Only PCS
Naess and Nyland^75^ 2013	Norway	188	48	44%	W/ first-ever cerebral infarction	NHP	6.0	Y		Y	
Bukan et al.^41^ 2014	Denmark	318	68^ǁ^	48.4%	ICU admission	SF-36, SF-12	90.0 d	Y	Both PCS	Y	Both PCS
Chapa et al.^64^ 2014	Canada, USA	693	72.0/68.5^‖‖^	37.8%	W/ atrial fibrillation	SF-36	3.5*	Y	Only Men PCS	Y	Only Men PCS
Kikkenborg Berg et al.^71^ 2014	Denmark	358	65.5	65%	ICD patients	EQ-5D VAS	1.3	NA		Y	
Ul-Haq et al.^21^ 2014	Scotland	5272	50	54.8%	Scottish population	SF-12	8.0	Y		Y	Varies by model
Wong et al.^60^ 2014	China	160	62^¶^	45%	Colorectal Ca	SF-6D	2.0	Y		Y	
Burns et al.^23^ 2015	Australia	14 019	73	91%	Older persons	SF-36	10.0	Y	Women both Men MCS	Y	Women both Men none
Gonzalez-Velez et al.^122^ 2015	Spain	412	84.7/87.5**	81.8%/81.9%**	People w/ dementia	EQ-5D	1.5	NA		Y N	Vary^¶¶^
Grincenkov et al.^92^ 2015	Brazil	1624	58	45%	On dialysis	SF-36	1.24^¶^	Y		Y	
Kielbergerová et al.^70^ 2015	Czech Republic	341	69	41.1%	Post-stroke Patients	SF-36	5.0	Y		Y	
Lizaur-Utrilla et al.^113^ 2015	Spain	1529	68.2^¶^	76.7%	Consecutive primary TKAs	SF-12	10.0	Y		Y	Only MCS
Pocock et al.^42^ 2015	Argentina, Belgium, Brazil, Denmark, Finland, France, Germany, Greece, Italy, Luxembourg, Mexico, Norway, Poland, Romania, Slovenia, Spain, Netherlands Turkey, UK, Venezuela	10 568	61.8	25.1%	Patients w/ non-fatal ACS	EQ-5D	1.0	NA		Y	
Bliemel et al.^109^ 2016	Germany	391	81	72%	W/ hip fracture	EQ-5D	1.0	Y		Y	
Hartog et al.^29^ 2016	Netherlands	184	79.2^¶^	70%	Older persons	RAND-36	1.0	N		N	
Martin-Lesende et al.^74^ 2016	Spain	83	81.6^¶^	42.2%	W/ HF and/or chronic lung disease	EQ-5D VAS	5.0	NA		Y	
Parlevliet et al.^44^ 2016	Netherlands	473	77.8	54.8%	Acutely admitted older patients	EQ-5D	1.0	Y		Y	Varies by model
Sexton et al.^103^ 2016	Ireland	362	63.2	59.4%	On haemodial.	EQ-5D VAS	3.4	Y		Y	
Perl et al.^102^ 2016	Australia, Belgium, Canada, France, Germany, Italy, Japan, New Zealand, Spain, Sweden, UK, USA	13 784	62	41%	On haemodial.	SF-12	0.92^¶^	NA		Y	Varies by model
Buecking et al.^111^ 2017	Germany	402	81	73%	W/ hip fracture	EQ-5D	1.0	Y		Y	
Cnudde et al.^110^ 2017	Sweden	42 862	67.7/75.8**	56.3%/47.0%**	W/ primary hip osteoarthritis	EQ-5D VAS	5.0	NA		Y	
Lahoud et al.^72^ 2017	USA	7056	51.7	59.6%	Primary cardiac prevention patients	SF-36	8.0	Y	Only PCS	Y	Only PCS
Pinheiro et al.^56^ 2017	USA	6290	NA	49.8%	W/ lung Ca	SF-36	2.0	NA		Y	
Ramos et al.^78^ 2017	Portugal	130	69	34%	W/ HF	SF-36	6.0	Y		Y	Varies by model
Reyes et al.^58^ 2017	USA	3734	NA	42%	W/ colorectal Ca	SF-12	5.0	Y		Y	
Stehlik et al.^80^ 2017	USA	200	63	27.0%	W/ HF	EQ-5D VAS	1.0	NA		Y	
Bakhru et al.^40^ 2018	USA	36	64.5	47%	ICU admission	SF-36	1.0	NA		N	
Higueras-Fresnillo et al.^34^ 2018	Spain	3922	71.8	56.4%	Older persons	SF-36	14.0^¶^	NA		Y	Varies by model
Jia et al.^30^ 2018	USA	105 473	74.6	58.0%	Older persons	SF-6D, EQ-5D	2.0	NA		Y	
Liira et al.^24^ 2018	Finland	3156	75 to 85^n^	52.4%	Older persons	15D	2.0	Y		Y	Varies by group^##^
Nater et al.^115^ 2018	USA	142	59.4	42%	Who had spinal decompressive Sx	SF-36, EQ-5D	1.0	Y	EQ-5D, SF-36 only PCS	Y	Only SF-36 PCS
Pinheiro and Reeve^55^ 2018	USA	535	75	50%	Lung Ca	SF-36	2.0	NA		Y	
Trajceska et al.^105^ 2018	Republic of Macedonia	162	56.2	47%	On haemodial.	SF-36	5.0	Y		Y	
Kikkenborg Berg et al.^63^ 2019	Denmark	998	63.8	20%	W/ ICD	SF-12, EQ-5D VAS	1.0	Y		Y	
Rosenberg et al.^32^ 2019	Canada	380	88.4	72%	Older persons	EQ-5D, EQ-5D VAS	1.5	Y		Y	Only VAS
van Veen et al.^85^ 2019	Netherlands	392	58	21%	W/ ICD	SF-36	7.0	N		N	
Case et al.^117^ 2020	USA	662	70^¶^	25.1%	W/ idiopathic pulmonary fibrosis	SF-12, EQ-5D, EQ-5D VAS	1.0	Y	All, SF-12 only PCS	N	
Kok et al.^108^ 2020	Canada	402	56.4	35.8%	W/ cirrhosis	EQ-5D VAS	0.5	Y		Y	
Frendl et al.^61^ 2020	USA	2425	73	0%	W/ prostate Ca	SF-36, VR-12	10.0	NA		N	
Phyo et al.^35^ 2021	Australia and USA	19106	74	56.4%	Older persons	SF-12	5.0	Y	Only PCS	Y	Only PCS***
Phyo et al.^36^ 2021	Australia and USA	19106	74	56.4%	Older persons	SF-12	5.0	Y		Y	
Singh et al.^37^ 2021	USA	13900	70.1	4%	Veterans	SF-12	7.0	NA		Y	Varies by model
Lou et al.^62^ 2022	Taiwan	1178	52.2	100%	W/ breast Ca and breast Ca Sx	SF-36	10.0	Y		Y	
Özyilmaz et al.^48^ 2022	Turkey	105	58.6	48.6%	Consecutive ICU patients	SF-12, EQ-5D	120 d	NA		Y	

*ACS* acute coronary syndrome, *AKI* acute kidney injury, *ALS* amyotrophic lateral sclerosis, *Ca* cancer, *CABG* coronary artery bypass graft surgery, *CAD* coronary artery disease, *CHF* chronic heart failure, *COPD* chronic obstructive pulmonary disease, *d* days, *Dx* diagnosis, *ED* emergency department, *EF* ejection fraction, *EQ-5D* EuroQol five dimensions questionnaire, *haemodial.* haemodialysis, *HF* heart failure, *HUI3* Health Utilities Index Mark 3, *ICD* implantable cardioverter defibrillator, *MCS* mental component score, *MDS-HSI* Minimum Data Set Health Status Index, *N* no, *NA* not available, *NHP* Nottingham Health Profile, *PAD* peripheral artery disease, *PCI* percutaneous coronary intervention, *PCS* physical component score, *pEQ-5D* predicted EQ-5D, *RAND-36* 36-Item Short Form Health Survey (SF-36), *Ref.* reference, *SF-12* SF 12-Item, *SF-6D* SF Six-Dimension, *SLE* systemic lupus erythematosus, *sympt.* symptoms, *Sx* surgery, *TKA* total knee arthroplasty, *UK* United Kingdom, *USA* United States of America, *VA* Veterans Affairs, *VAS* visual analogue scale, *VR-12* Veterans RAND 12 Item Health Survey, *W/* with, *Y* yes

*Mean in years

^†^Female

^‡^Follow-up. Time is provided in years unless otherwise specified

^§^All SF instruments include physical and mental component scores

^‖^Diabetic/non-diabetic

^¶^Median

^#^Hospitalized/not hospitalized

**Alive/deceased

^††^Treated with sunitinib/treated with interferon alfa

^‡‡^Colorectal cancer/lung cancer

^§§^Person-years

^‖‖^Women/men

^¶¶^Vary by the presence of depressive symptoms. Score on the Cornell Depression Scale for Dementia <6 or ≥6

^##^Eight groups were included in this study: home-dwelling cardiovascular patients; former businessmen; home-dwelling people with dementia; spousal caregivers of people with dementia; hospitalized patients with delirium; nursing home residents; older people suffering from loneliness; population sample

***Adjusted by age predicted fatal cardiovascular disease, fatal myocardial infarction, and fatal stroke. Adjusted by age, and other sociodemographic and clinical characteristics, predicted fatal myocardial infarction

### Overview of Included Articles

Articles were published between 1997 and 2022, with over one-third (*n*=42) from the United States of America (USA). Nearly a quarter (*n*=24) pertained to people with cardiovascular diseases (CVD), followed by kidney disease (*n*=17), with seven pertaining to the general population. The median number of participants was 879, ranging from 36 to 105,473. Nearly three-quarters of studies (*n*=81) used at least one of the RAND Corporation QoL surveys: RAND-36/36-Item Short Form Survey (SF)-36, 12-Item SF Survey (SF-12), Veterans RAND 12-Item Health Survey (VR-12), and SF Six-Dimension (SF-6D). Nearly a quarter of studies (*n*=26) used a utility instrument, predominantly the EQ-5D (*n*=19); SF-6D (*n*=5); the Health Utilities Index Mark 3 (HUI3) (*n*=2); the 15-Dimensional instrument (15D) (*n*=1); and the Minimum Data Set Health Status Index (MDS-I) (*n*=1). Two studies used two utility instruments, the EQ-5D and the SF-6D (Table 2). There was variation of timeframes employed for mortality predictions, reported as total, median, and mean follow-up as well as person-years, and ranged from a median follow-up of 18.5 days to a median follow-up of 14 years.

### Prediction of Mortality by Instrument

Two-thirds of studies (*n*=72) undertook univariate analysis and all but one (*n*=109), multivariate analysis, with all identified instruments employed for each. Cox proportional hazards regression was the most frequently used statistical method for both univariate and multivariate analyses (*n*=43 and 79 respectively), followed by logistic regression (*n*=17 and 25 respectively).

HRQoL predicted mortality in all but five univariate analyses, of which two employed utility instruments (EQ-5D and MDS-I). Among studies that used the SF-36 and/or SF-12 and found a univariate association (*n*=37 and *n*=15, respectively), over half of each (*n*=21, *n*=9, respectively) observed a relationship between both the physical component score (PCS) and mental component score (MCS) and mortality, including one study that used both instruments. Two studies employing the SF-36 found variation in results by sex (Table 2).

In multivariate analyses, most studies (*n*=100) found HRQoL predicted mortality regardless of the instrument employed. In the nine studies that did not find an association, three employed utility instruments (MHS-I, EQ-5D, and SF-6D). Instruments for which there was always an association included two utility instruments (HUI3, 15D) and the NHP (Table 2). Among studies that used either the SF-36 or the SF-12 and found an association (*n*=53 and *n*=19, respectively), over half (*n*=29 and *n*=12, respectively) observed a relationship for both the PCS and MCS and mortality, including one study that used both instruments. There were eight studies using the SF-36 that reported variation in their results by sex (*n*=2); age (older vs younger patients) (*n*=1); diabetes status (yes/no) (*n*=1); cause of death (all-cause vs CVD); and model tested (*n*=1); with inclusion of depressive symptoms (*n*=2); and with different confounders (*n*=1). Four studies using the SF-12 reported variation by model tested with inclusion of depressive symptoms; body mass index (BMI); and sociodemographic and clinical characteristics; and by changes in HRQoL over time.

### Prediction of Mortality by Population

**Fig. 2:**
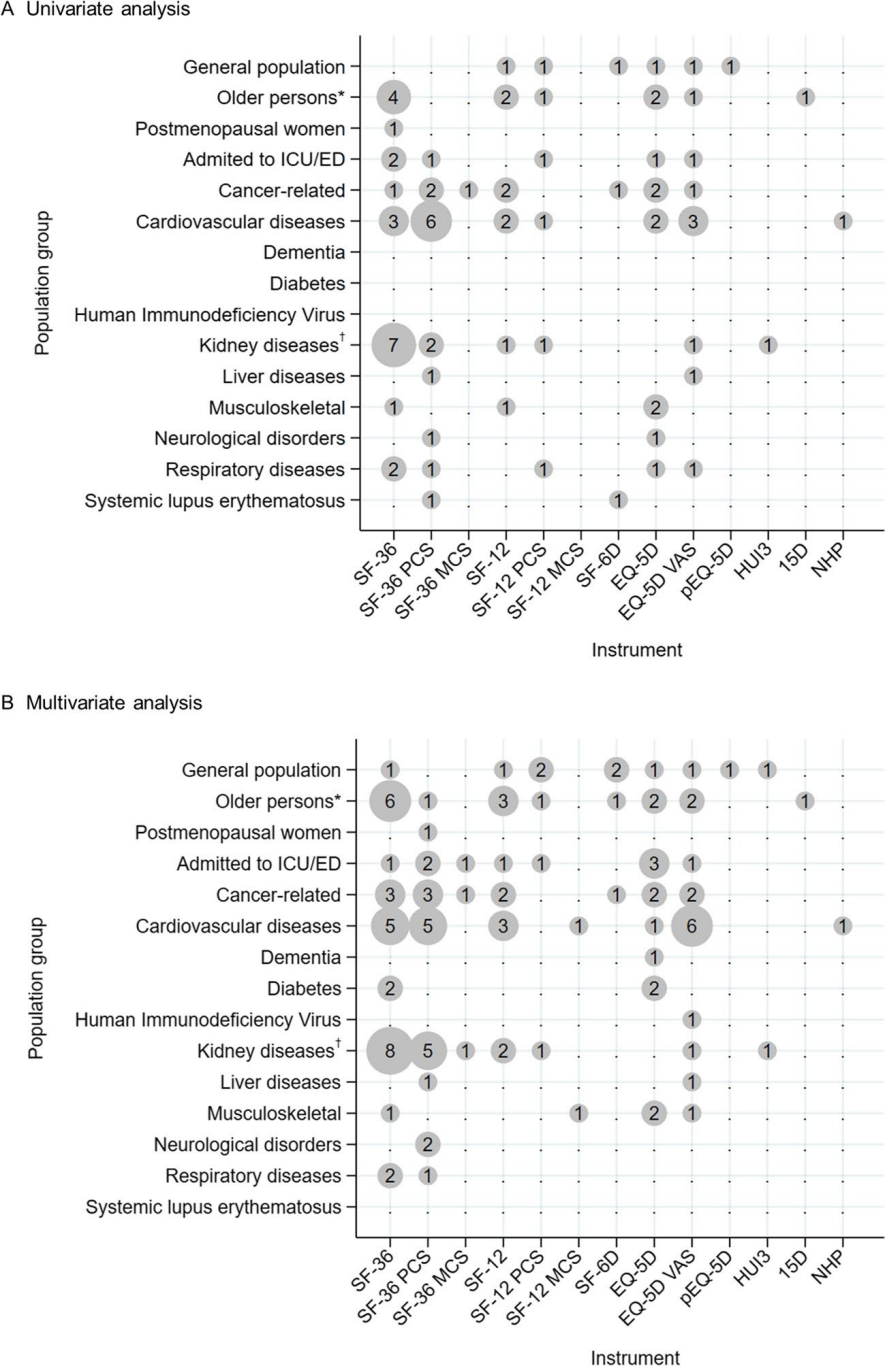
Bubble plots of univariate and multivariate predictions of mortality by study population and instrument employed, for identified relationships. *Legend*: The size of a bubble is proportional to the number of prediction of mortality in the population group and instrument corresponding to the bubble coordinates, reported in the articles included in the review. 15D, 15-Dimensional instrument; ED, emergency department; EQ-5D, EuroQol five dimensions questionnaire; HUI3, Health Utilities Index Mark 3; ICU, intensive care unit; MCS, mental component score; NHP, Nottingham Health Profile; PCS, physical component score; pEQ-5D, predicted EQ-5D; SF-12, Short Form Health Survey 12-Item; SF-36, SF 36-Item; SF-6D, SF Six-Dimension; VAS, visual analogue scale. *Including veterans. ^†^Including dialysis and haemodialysis.

There were seven general population studies^16–22^ conducted over periods of 1 to 10 years. Three studies provided univariate results, with HRQoL predicting mortality in each^17,20,21^ (Fig. 2A). The results were consistent in multivariate analyses, although in one study,^21^ inclusion of BMI led to variation. HRQoL predicted mortality in the four studies that undertook multivariate analysis only (Fig. 2B).

Fifteen studies were conducted in persons 60 years or older,^23–37^ and another included veterans 50 years or older (average 67.8 years).^38^ Studies were undertaken over periods of 1 year (total follow-up) to 14 years (median follow-up). Participants included the following: veterans enrolled in general internal medicine clinics; older persons with no specific conditions, with one or more chronic diseases, and admitted to a nursing home; a non-institutionalized population; and people receiving home-based primary care with presence of a frailty syndrome or multiple comorbidities. Of 11 studies that reported univariate analyses,^23–25,27–29,31,32,35,36,38^ all except one^29^ found HRQoL predicted mortality. However, in one study,^23^ results varied by sex (Fig. 2A). Multivariate analyses were undertaken in all studies, with HRQoL predicting mortality in all analyses except for persons admitted to a nursing home,^29^ as in the univariate analysis (Fig. 2B and Table 2). In five studies, results were found to vary by sex^23^; model tested^31,35^; model and cause of death (all-cause vs CVD)^34^; and eight heterogeneous samples of older persons included.^24^

A single article reported on postmenopausal women.^39^ The study was undertaken over a period of 3 years, with HRQoL predicting mortality in both univariate and multivariate analyses (Fig. 2 and Table 2).

#### Clinical Sub-populations

Nine articles reported on people admitted to intensive care units or emergency departments who were followed up after discharge over periods of 90 days to 7 years.^40–48^ All except four^40,42,43,48^ presented univariate analysis results with HRQoL predicting mortality irrespective of instrument employed (Fig. 2A). In multivariate analyses, mortality was predicted by HRQoL in all but one study.^40^ Further, in a study that assessed HRQoL 3 months after admission,^44^ mortality was predicted in a model adjusted by age and sex and in the fully adjusted model (including age, sex, and other confounders: delirium, Mini-Mental State Examination score, baseline Katz activity of daily living score, number of geriatric conditions, and Charlson Comorbidity Index score). However, when HRQoL was assessed 12 months after admission, mortality was predicted only in the model adjusted by age and sex (Fig. 2B and Table 2).

Fourteen articles reported on people with cancer^49–62^ over periods of 18.5 days (median follow-up) to 10 years (total follow-up). Five studies did not provide a univariate analysis.^50,52,55,56,61^ In all analyses, HRQoL predicted mortality irrespective of instrument employed (Fig. 2A). In multivariate analyses, mortality was predicted by HRQoL in all but one study.^61^

Twenty-four articles reported on people with a CVD^63–86^ over periods of 30 days to 8 years. Univariate analysis was undertaken in all but six studies,^67,69,71,74,80,82^ with HRQoL predicting mortality in all but two.^83,85^ Two more studies employed two instruments and found associations for each^63,73^ (Fig. 2A). In another study, results varied by sex, with none of the SF component scores found to predict mortality in women while, in men, the PCS was found to be a significant predictor.^64^ All but one article^83^ reported multivariate analyses. With the exception of two studies,^69,85^ mortality was predicted by HRQoL in all the remaining studies (including two that used two instruments^63,73^) (Fig. 2B). Three articles reported variation in results with HRQoL no longer predictive of mortality after the inclusion of depressive symptoms.^66,78,84^ Differences by sex were captured in one study^64^ and differences by age in another.^67^

Four articles reported on people with diabetes^8,87–89^ conducted over periods of 5 to 10 years. No study reported univariate results, with HRQoL predicting mortality in all multivariate analyses (Fig. 2).

Seventeen articles reported on people with a kidney disease involving people on dialysis or haemodialysis^90–106^ over periods of 6 months to 6 years. Eleven studies found HRQoL predicted mortality based on univariate analysis, including two studies that used two instruments.^97,100^ The other six studies did not provide univariate results.^90,93,95,99,102,106^ All 17 studies found HRQoL predicted mortality in multivariate analyses. Variation was found in a study that stratified people with and without diabetes^98^ and by changes in HRQoL over time^102^ (Fig. 2 and Table 2).

Only two studies were conducted in people with a liver disease^107,108^ over periods of 6 months and 3 years, with HRQoL found to predict mortality in univariate and multivariate analyses in both (Fig. 2).

Five studies were conducted in people with a musculoskeletal condition^109–113^ over periods of 1 to 10 years using different instruments. HRQoL was found to predict mortality through univariate analysis in four studies; the fifth did not provide results.^110^ In multivariate analyses, HRQoL was found to predict mortality in all studies regardless of instrument (Fig. 2 and Table 2).

Two articles reported on people with a neurological condition^114,115^ over periods of 1 and 4 years. In Nater et al.,^115^ the SF-36 PCS and the EQ-5D predicted mortality in univariate analysis, but only the SF-36 PCS in multivariate analysis. In del Aguila and collaborators,^114^ the SF-36 did not predict mortality in univariate analysis, but the PCS did predict mortality in multivariate analysis.

Six articles reported on people with a respiratory disease^116–121^ over periods of 30 days to 5 years using a variety of instruments. Four of five studies that provided results of univariate associations found that HRQoL predicted mortality, including a study that employed three instruments^117^ (Fig. 2A). In multivariate analyses, mortality was predicted by HRQoL in half the studies^118,120,121^ (Fig. 2B and Table 2).

Three more articles reported on people with dementia,^122^ adults infected with human immunodeficiency virus (HIV),^123^ and patients with systemic lupus erythematosus (SLE).^124^ The first two studies did not provide results from univariate associations,^122,123^ while the third found an association between HRQoL and mortality (Fig. 2A). Multivariate analyses found that HRQoL only predicted mortality in people with dementia with limited depressive symptoms (<6 on the Cornell Depression Scale for Dementia). HRQoL also predicted mortality in those infected with HIV, but not in people with SLE (Fig. 2 and Table 2).

## DISCUSSION

This scoping review is the first to provide an overview of the literature on generic HRQoL scores as predictors of mortality across general populations and clinical sub-populations. Among the 110 studies mapped, nearly a quarter included people with cardiovascular diseases followed by people with kidney disease, dialysis, and haemodialysis. There were no studies investigating diagnosed mental health conditions. Eleven instruments were employed, the SF-36 the most used. Most studies assessed relationships through multivariate analysis using Cox proportional hazards models. For some studies using the SF-36 and SF-12, only the PCS or MCS was found to be associated with mortality in univariate and/or multivariate analyses.

Through this review, a consistent relationship between low HRQoL scores and mortality was observed independent of the generic instrument employed, with consistent findings when multiple instruments were employed.^17^ The relationship is clearest in the univariate analyses, but also observed in most multivariate analyses. Our finding that lower HRQoL scores are associated with mortality in general non-patient populations is consistent with results from Phyo and collaborators.^5^ We also found this relationship held in all clinical populations investigated in either univariate or multivariate analysis.

We acknowledge that there is no proposed mechanism through which HRQoL *causes* death. However, as it is not possible to perfectly measure the degree to which each person’s disease or multiple diseases in combination affect their mortality risk, HRQoL as a holistic assessment of health status appears useful for this purpose. Unadjusted results are of inherent value for standalone screening and monitoring. Additionally, disease-specific HRQoL instruments are not required as predictors of survival^125,126^ within the populations covered by this review.

There is some evidence of an attenuation of effect between univariate and multivariate analyses, showing the impact of specific health-related factors on the strength of association. For example, in the only two studies including people with CVD that adjusted for depressive symptoms,^66,78^ neither component score of the SF-36 was significantly associated with survival when depressive symptoms were included in their models. This review also identified the following variables as important to the assessment of an association: sex, cause of death, and the timepoint of assessment (e.g., 3 or 12 months post-admission to intensive care). However, multivariate analysis is not considered as useful for a simple holistic assessment of mortality risk.

Findings also support the importance of choice of timeframe in assessments. For instance, in studies employing short timeframes (up to 30 days), associations between HRQoL and mortality were found for conditions with an anticipated short life expectancy, i.e., renal cancer^50^ and terminal cancer^54^ but not otherwise.^83,116^ In contrast, associations between HRQoL and longer-term mortality were found in study populations with a range of different health conditions,^62,65,79,82,117–121^ suggesting that generic HRQoL measures can also be indicative of longer-term mortality risk.

Our findings identified that, when the association between mortality and HRQoL was with either the PCS or MCS of an SF survey, the PCS was more frequently associated with mortality. These findings also highlight the likely importance of different factors within holistic assessments across conditions, and the potential benefits of a single overall score as for multi-attribute utility assessments. Extending Clarke and colleagues’ findings on cumulative and/or extreme problems,^8^ we postulate a detailed understanding of condition-specific cumulative and/or extreme problems associated with mortality (and other events) may provide useful information for screening and/or monitoring within the clinical context.^32,102^

Clarke and colleagues^8^ also raised a question about the predictive ability of utility instruments other than the EQ-5D in people with type 2 diabetes; our review suggests this is a moot point as no other utility instruments have been employed in this sub-population. However, relationships between EQ-5D utilities and mortality were identified in all the study populations investigated, with relationships for utilities assessed with other instruments found in five of six study populations investigated.

The results of this study are subject to the review design, the review question, and specific limitations of the included studies. As a scoping review, the quality of studies and appraisal of methodological risk of bias were not undertaken.^12^ Likewise, due to the exploratory design of this review, the heterogeneity in the instruments used for the assessment of HRQoL, and diversity of study populations assessed, we did not report HRQoL scores. We undertook high-level comparisons only and no summary statistics were assessed. Even so, we have identified a general association between HRQoL scores and mortality, regardless of the instrument or analysis employed, for both the general population and most sub-populations with specified physical health conditions and/or risk factors.

## CONCLUSIONS

HRQoL was found to be an indicator of mortality risk in the general population and most clinical sub-populations, independent of the generic instrument employed. However, no studies investigated the relationship between HRQoL and people living with diagnosed mental disorders. This is an important gap, as rates of poor physical health and mortality are much higher in people with mental disorders,^127^ especially those with severe mental illness,^128,129^ compared to the general population. In the clinical context, HRQoL assessment may be useful for screening and/or monitoring purposes to understand how people perceive their health and well-being and as an indicator of mortality risk, encouraging better-quality and timely patient care to support and maximize what may be a patient’s only modifiable outcome.

### Supplementary Information

ESM 1 (DOCX 21 kb)
