# Supplementary material for: Health-Related Quality of Life Scores and Values as Predictors of Mortality: A Scoping Review
Source: J Gen Intern Med. 2023 Aug 31;38(15):3389–405. doi: 10.1007/s11606-023-08380-4 (PMC10682357; doi:10.1007/s11606-023-08380-4)
Supplement: Supplementary file 1 — (DOCX 21 kb) [file 11606_2023_8380_MOESM1_ESM.docx]

**Supplementary Table 1** PubMed Search Strategy

| **Search** | | **Term** |
| --- | --- | --- |
| #1 |  | “utility value”[Text Word] |
|  | OR | “utility values” [Text Word] |
|  | OR | “utility score” [Text Word] |
|  | OR | “health state”[Text Word] |
|  | OR | “health related quality of life”[Text Word] |
|  | OR | “health-related quality of life”[Text Word] |
|  | OR | HRQoL[Text Word] |
|  | OR | AQoL[Text Word] |
|  | OR | EQ-5D[Text Word] |
|  | OR | SF*[Text Word] |
|  | OR | HUI[Text Word] |
|  | OR | 15D[Text Word] |
| #2 |  | predictor*[Text Word] |
|  | AND | mortality[Text Word] |
|  | OR | mortality[MeSH Terms] |
| #3 | #1 AND #2 | |

*Filters activated: Humans, English, Adult: 19 + years*
